# Response to “ChatGPT-polished scientific writing and artificial intelligence detection: Cohorts, baselines, fairness”

**DOI:** 10.1016/j.jdin.2025.11.005

**Published:** 2025-11-13

**Authors:** Evelyn Shue, Neil K. Jairath, Gangqing Hu

**Affiliations:** a Department of Biology, Duke University, Durham, North Carolina; b Department of Medicine, Indiana University, Indianapolis, Indiana; c Department of Microbiology, Immunology & Cell Biology, West Virginia University, Morgantown, West Virginia

**Keywords:** ChatGPT, ethics, fairness, manuscript editing, medical education, non-native English writers

*To the Editor:* We appreciate Du and Koga’s interest^1^ in our work and are grateful for the opportunity to respond. Our previous work investigates the trend of artificial intelligence (AI) assistance in editing dermatology manuscripts and the impact of AI-polishing on credit attribution when assessed by GPT detectors.^2^ We compare 2020 and 2024 the Journal of the American Academy of Dermatology letters by authors from 3 non-English–speaking Asian countries to those by US authors and report 2 findings. First, for non-native English authors, the 2024 letters exhibit a higher chance of being flagged as AI-assisted than the 2020 letters. Second, after AI-polishing the letters simply to improve readability, 75% to 85% of them were marked as AI-generated by GPT detectors regardless of years or authors’ language backgrounds.^2^

Du and Koga^1^ comment on the imperfection of using institutional affiliations as a proxy of English “nativeness”. While publications usually do not disclose authors’ language backgrounds, affiliations are used as a first-order approximation in which we require all affiliations be from the same country to minimize confounding effects. We recommend interpreting “nativeness” from the perspective of language use in daily practice and academic activities. For example, while some US authors may come from non-English–speaking environments, their residency training and clinical practice are conducted in English. We agree that expansion into more diverse language backgrounds would help validate generalization. Indeed, a similar work published concurrently with ours,^3^ although not on dermatology, includes 5 Anglosphere countries and 18 non-English–speaking countries. Consistent with our findings, their work also identifies AI-polishing as a risk factor for human-authored drafts being miscredited as AI-generated.^3^

Our work measures changes and includes 2 types of baseline controls: the 2020 letters for studying the trend of AI-assisted writing and the original letters for evaluating the risk of AI-polishing.^2^ By focusing on the baselines from the 2020 letters, Du and Koga find that the original letters from non-native authors are flagged less often as “human (high)” than the US authors, interpreting this as a bias against non-native authors^1^ and confirming an earlier finding on a different topic.^4^ However, GPT-1 and GPT-3 were released in June 2018 and June 2020, respectively. Therefore, we remain cautious about their interpretation because the possibility of a need-driven adaptation to these models by non-native authors as early as 2018 to 2020 cannot be excluded.

We agree with Du and Koga^1^ that content other than tools are the focus of human reviewers. A simple disclosure may be justified if the AI usage is to improve readability. However, the use of AI for content generation—such as generating an initial draft from an outline—warrants heightened scrutiny or even discouragement. Such practice blurs the line between human and unethical AI authoring, is error-prone, and may jeopardize long-term innovation.^5^ As AI assistance becomes increasingly prevalent in scientific writing, much like disclosures of AI usage, authors who involve no AI might consider adding a declaration of non-AI assistance upon manuscript submission.^3^ This is the case of this letter, which echoes an ongoing practice in some journals like the Journal of the American Academy of Dermatology.

### Declaration of generative AI and AI-assisted technologies in the writing process

None.

## Conflicts of interest

None disclosed.
